# Acute Thrombocytopenia, Leucopenia, and Multiorgan Dysfunction: The First Case of SFTS Bunyavirus outside China?

**DOI:** 10.1155/2011/204056

**Published:** 2011-10-12

**Authors:** Srdjan Denic, Joumana Janbeih, Suresh Nair, Walter Conca, Waheed Uz Zaman Tariq, Suhail Al-Salam

**Affiliations:** ^1^Department of Internal Medicine, Faculty of Medicine and Health Sciences, UAE University, P.O. Box 17666, Al Ain, UAE; ^2^Department of Medicine, P.O. Box 1006, Al Ain Hospital, Al Ain, UAE; ^3^Department of Pathology, Tawam Hospital, P.O. Box 15258, Al Ain, UAE; ^4^Department of Pathology, Faculty of Medicine and Health Sciences, UAE University, Al Ain, UAE

## Abstract

We report a 57-year-old man with acute thrombocytopenia, leucopenia, and multiorgan dysfunction. Patient was from North Korea and was temporarily working in Dubai, United Arab Emirates, when he fell ill in March 2009. At the same time and unknown to us, many patients with similar clinical manifestations were admitted to hospitals in China. The Chinese cases—identified between March and July 2009—were recently reported to have been infected with a tick-born strain of bunyavirus, a new disease. The virus infection was documented in patients from central China and the region that shares the border with North Korea. The clinical manifestations, the time of disease onset, and geographical link of the patient with the region in which the disease is endemic suggest that the patient had SFTS bunyavirus infection.

## 1. Introduction

Severe fever and thrombocytopenia syndrome (SFTS) is a newly identified disease in China that is caused by a strain of bunyavirus. The disease is characterized by fever, thrombocytopenia, leucopenia, bleeding, and multiorgan dysfunction and has 30% mortality rate. It is transmitted by a tick and has not been reported outside China. The clinical and epidemiological description of the disease in English literature is sparse [1].

## 2. Case Presentation

**Figure 1:**
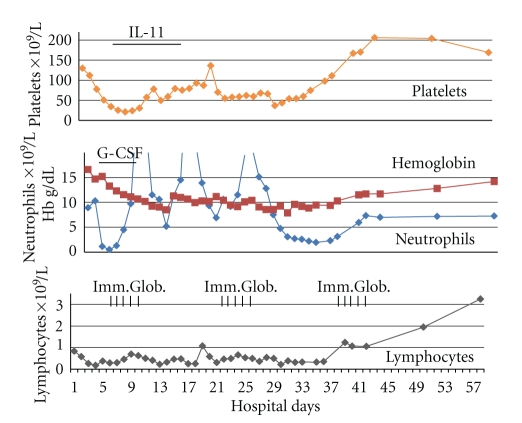
Blood cell counts during hospitalization. Abbreviations: IL-11: interleukin-11; G-CSF: granulocyte-colony stimulating factor; Imm.Glob: human immunoglobulins.

**Figure 2:**
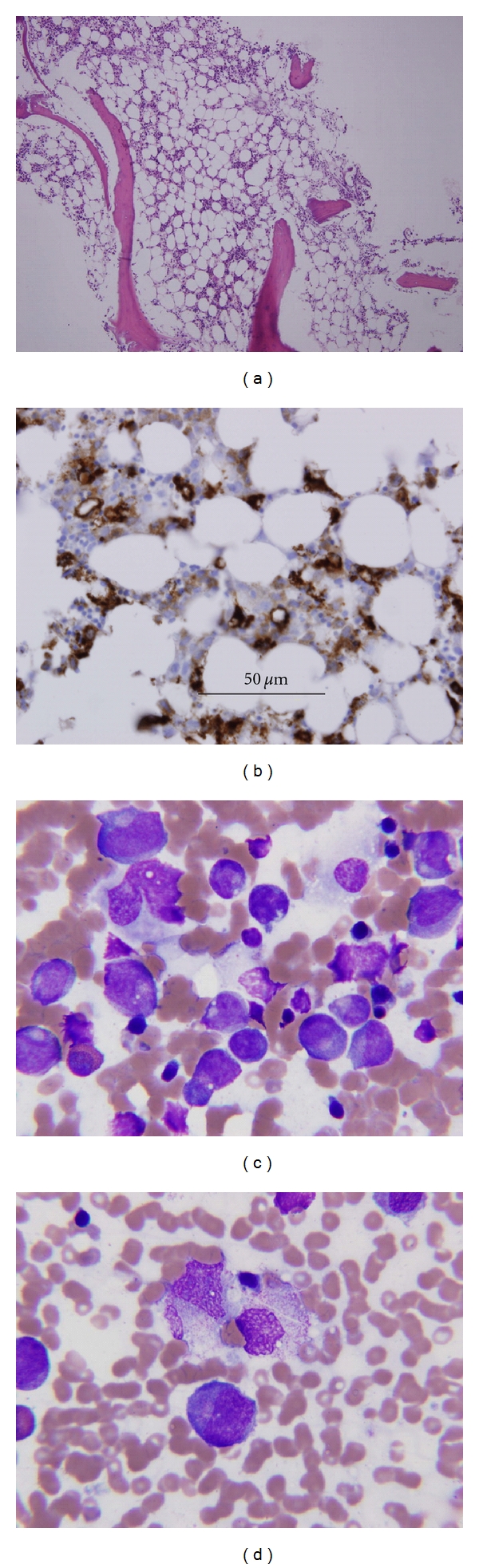
Bone marrow study: (a) mild hypocellularity. (b) Positive histiocytes stain for CD68 marker. (c) Myeloid cell line composed mostly of promyelocytes with prominent Golgi apparatus and absent neutrophils and metamyelocytes (maturation arrest). (d) Histiocytes and promyelocyte (center) with a rare histiocyte showing hemophagocytosis.

A previously healthy 57-year-old North Korean male working in Dubai, United Arab Emirates (UAE), developed an acute hemorrhagic stroke in March 2009. There was no history of trauma, abuse of tobacco or alcohol, exposure to toxic fumes or dust, or traveling outside UAE during the last 12 months. On physical examination he was drowsy and moving all four extremities. Temperature was normal, blood pressure 220/116 mmHg, and heart rate 98 per minute. CT scan of the brain showed thalamic hemorrhage with blood extension into the ventricular system and CT angiography showed no evidence of an aneurysm. The patient was intubated, chest tube was placed because of a left pneumotorax at the time of intubation. Blood pressure required control with labetolol only for initial few days. On admission, hemoglobin was 16.6 g/dL, neutrophils 8.9 × 10^9^/L, lymphocytes 0.8 × 10^9^/L and platelets 130 × 10^9^/L. Within 48 h, patient developed fever, severe neutropenia, thrombocytopenia, and a more profound lymphocytopenia (Figure 1) and skin and lung bleeding. Coagulation tests were normal. Bone marrow examination findings are shown in Figure 2. Toxic screen was negative. The flow cytometry disclosed normal CD8 and low CD3, CD4, and CD19 cell counts; IgG level was decreased. Pneumonia and *K. pneumoniae* sepsis developed but there was no evidence of disseminated intravascular coagulation, hemolysis or renal impairment. Treatment with Tazocine, immunoglobulins, granulocyte-colony stimulating factor, steroids, and interleukin-11 was commenced. CT scan of the chest showed bilateral pneumothorax, lung bullae, and consolidation; however, blood oxygenation of the patient was relatively good throughout hospital stay and serum activity of alpha-1-antitrypsin was normal. Tests for systemic lupus erythematosus, antiphospholipid syndrome and c- and p-ANCA were negative. Tests for HIV1, HIV2, cytomegalovirus, Epstein-Barr virus, herpes simplex virus, and Mycoplasma pneumoniae infection were all negative on admission and three weeks later. Infection with Legionella pneumophila was excluded with negative urine test for its antigen. During the third and fourth weeks of hospitalization the patient had hepatitis and myositis. Acute hepatitis A, B, and C infections were excluded with repeated serological tests and the tests for Dengue and Crimean-Congo hemorrhagic fever (CCHF) were negative. In the fourth week of hospital stay, patient had complete quadriplegia with preserved sensation of pain and touch. Paralytic ileus developed due to autonomic neuropathy and was treated conservatively. Electroneuromyographic study disclosed evidence of axonal motor radiculoneuropathy and cerebrospinal fluid examination showed findings consistent with aseptic meningitis. Repeated doses of immunoglobulin were given and the patient began to recover. On the 60th hospital day, his motor power was 4/5 and improving, all cell counts and biochemical tests were normal, and he was discharged home.

## 3. Discussion

Clinical manifestations in our patient are best explained with a systemic viral infection and, on epidemiological and clinical grounds, it likely was SFTS bunyavirus. 

### 3.1. Epidemiology

Our patient had clinical manifestations similar to those in patients with SFTS bunyavirus infection. The onset of his disease (March 2009) occurred simultaneously with the onset of epidemic of SFTS bunyavirus infection in China (March–July 2009). In addition, some of the Chinese patients were from the Liaoning province that share border with North Korea [1]. Thus clinical presentation and spatiotemporal epidemiology of patient's illness suggest that he had SFTS bunyavirus infection. Our patient did not report history of tick bite but the absence of history of tick bite is common in patients with diseases transmitted by ticks. The absence of history of recent travel to home country is a missing link in this case. Incubation period and duration of the persistence of the virus in the body is yet unknown. Although human to human transmission of SFTS was not reported previously, it cannot be excluded altogether; other tick-borne bunyaviruses may be parenterally transmitted via body fluids and tissue, in settings that provide close contacts of people. Our patient resided abroad with his fellow countrymen in a densely populated environment created for temporary workers in which men periodically come from and go to their home country. In such environment, the transmission of infectious agent by direct contact may be possible; in the past, this occurred with CCHF in Dubai, another bunyavirus infection [2]. Similarly, import of infected tick from home country cannot be excluded as arthropod-borne diseases spread more readily within the deprived populations [3].

### 3.2. Clinical Manifestations

The full spectrum of clinical manifestations of SFTS is not well documented in English language literature. *Cerebral hemorrhage* in the patient could have been due to a virus-induced vasculitis [4–6]. Vasculitis associated with ANCA autoantibodies was excluded with negative tests [7]. CT angiography may not detect small vasculitic changes and the absence of aneurysm does not exclude vasculitis. Hypertension in our patient is an unlikely cause of cerebral hemorrhage and more likely an immediate reaction to an increased intracerebral pressure from bleeding; patient subsequently neither had hypertension nor the signs of its preexistence. *Transient thrombocytopenia, neutropenia, and lymphocytopenia *were most likely the consequence of virus infection as well because evidence does not support other possible causes: multisystem diseases, drugs, and toxins [8]. Bone marrow findings are also consistent with viral infection (Figure 2). Several viral infections could produce lymphocytopenia and low IgG level via “cytokine storm” that is also associated with a depression of granulocyte and monocyte counts, both decreased in our patient [9–14]. Bacterial sepsis in the patient could produce neutropenia and thrombocytopenia but this is excluded by the presence of cytopenias prior to the development of bacterial infection. Similarly, stroke-induced immunosuppression may produce neutropenia but thrombocytopenia, myositis, hepatitis and Guillain-Barre neuropathy are not part of this syndrome [15]. *Acute hepatitis and myositis* could be caused by several types of viruses but repeated tests for those agents were negative. The type and doses of medications which were given to the patient are not strongly associated with the development of hepatitis, and myositis. Similarly,* K. pneumoniae* infection is unlikely a cause of myositis [16–18]. Further, there was no evidence of primary multisystem diseases that could cause hepatitis and myositis. Consequently, unidentified virus becomes the most likely etiology of hepatitis and myositis. *Acute motor axonal neuropathy type of Guillain-Barré syndrome* was present in our patient and was related in the past to several bacterial and viral infections. However, none of our repeated tests were positive for these infections. Further, Guillain-Barré syndrome was reported in patient with suppressed immune function by drugs, HIV, and CMV but those causes were excluded in our transiently immunocompromised patient [19]. 

In summary, clinical manifestations in our patient are best explained by systemic viral infection. We did not find in literature such a combination of manifestations in a single patient. The presence of acute thrombocytopenia, neutropenia, lymphocytopenia, bleeding, and multiorgan dysfunction was all reported in patients with SFTS bunyavirus infection [1]. The absence of high fever on admission argues against SFTS in our patient. However, temperature increased once thrombocytopenia and leucopenia developed despite administration of steroids.

## 4. Conclusion

Clinical manifestations and spatiotemporal epidemiology of the disease suggest diagnosis of SFTS bunyavirus infection.
